# Effect of obesity and exercise training on circulating lipids in American Indian adolescents

**DOI:** 10.1371/journal.pone.0338547

**Published:** 2025-12-16

**Authors:** Charles F. Hojjat, April M. Teague, Mary A. Tullier, Jennifer Q. Chadwick, Kenneth C. Copeland, Kevin R. Short

**Affiliations:** 1 College of Medicine, University of Oklahoma Health Campus, Oklahoma City, Oklahoma, United States of America; 2 Department of Pediatrics, University of Oklahoma Health Campus, Oklahoma City, Oklahoma, United States of America; 3 Choctaw Nation of Oklahoma, Durant, Oklahoma, United States of America; Murdoch Children's Research Institute, AUSTRALIA

## Abstract

Childhood dyslipidemia is predictive of future cardiometabolic disease. We determined the impact of obesity (Ob) and an exercise program on lipids in American Indian adolescents. This is a secondary analysis from a previously reported trial designed to determine the impact of financial incentives on exercise behavior in American Indian adolescents who were tested in Southeast Oklahoma between June 2013 and January 2017. We performed a cross-sectional comparison of fasting lipids in participants with Ob (n = 74) and a group with normal weight (NW, N = 39) at baseline prior to the exercise intervention. Measurements were repeated for the Ob group after they completed a 16-week exercise program (n = 53). Compared to the NW group, the Ob group at baseline had higher triglycerides (median [interquartile range]: 1.02 [0.67, 1.73] vs. 0.55 [0.40, 0.74] mmol/L), oxidized high density lipoprotein (HDL) (393 [311, 511] vs. 316 [281, 360] ng/mL), oxidized low density lipoprotein (LDL) (49.3 [38.0, 58.6] vs. 38.8 [29.8, 43.4] U/L), and 4 out of 11 targeted fatty acids measured, while HDL-C (mean ± SD: 0.61 ± 0.18 vs. 0.89 ± 0.20 mmol/L) was lower, all p < 0.001. Fatty acid ratios suggested that hepatic lipid metabolism was altered in the Ob group, with higher stearoyl desaturase, and lower elongase and delta-6 desaturase activities. Aerobic fitness increased 10% in the Ob group but among the lipids measured, only elaidic and myristic acid declined in response to exercise. This study is among the first to report that several plasma lipids were altered in American Indian adolescents with Ob and only minor changes occurred following an exercise program. These findings should be interpreted as exploratory and hypothesis-generating, given the study’s observational and secondary analysis design, but highlight the elevated cardiometabolic disease risk in this understudied population and the need for effective prevention and intervention strategies.

## Introduction

Lipids are involved in many essential processes, such as controlling what goes into and out of cells, energy storage, and vitamin absorption. Additionally, many lipids are markers of cardiometabolic disease. For example, in adults, increased serum saturated fatty acids are associated with higher risk of developing type 2 diabetes (T2D) and cardiovascular disease [1]. Furthermore, elevated total cholesterol, low-density lipoprotein cholesterol (LDL-C), and triglycerides (TG) are associated with an increased risk of pre-diabetes and T2D [2]. With obesity, impaired hepatic fatty acid oxidation results in lipid accumulation and redox imbalance. Lipid accumulation promotes the development of steatotic liver disease and insulin resistance through decreased expression of Kruppel-like transcription factor 16 [3]. In response to insulin resistance and obesity, expression of the Cyclin-Dependent Kinase Inhibitor 2C growth regulator leads to impairment of lipid storage, which can contribute to increased circulating non-esterified fatty acids (NEFA) in people with T2D [4,5]. Adults with overweight or obesity typically have dyslipidemia, while weight loss can promote a reduction in LDL-C and increase in HDL cholesterol (HDL-C) [6].

Obesity and low aerobic fitness in childhood elevates the risk for cardiometabolic disease during adulthood [7]. There are fewer studies of obesity-associated dyslipidemia in children than adults. It was shown that the concentrations of the omega-6 fatty acids arachidonic acid and linoleic acid were increased in children with obesity compared to those with normal weight [8]. Another study demonstrated that adolescents with obesity had higher LDL-C and lower plasma alpha-tocopherol [9]. Additionally, obesity is the greatest risk factor for decreased HDL-C particle size in adolescents [10]. We previously reported that oxidized LDL (oxLDL) and oxidized HDL (oxHDL) were increased in adolescents with obesity and T2D, but not in adolescents with obesity without T2D [11]. Circulating lipids are also affected by acute and chronic physical activity. For example, a study of children with overweight or obesity who were 5–10 years old reported that habitual level of light physical activity was positively related to HDL-C concentration, more so than moderate to vigorous physical activity [12].

American Indian children and adolescents have high rates of obesity [13], which contributes to their higher incidence of T2D compared to most other racial and/or ethnic groups in the United States [14]. We completed an exercise intervention with American Indian adolescents who were considered at increased risk for cardiometabolic disease because they had excess body weight and were not meeting current guidelines for physical activity [15]. We previously reported that in comparison to a control group with normal weight, participants with overweight or obesity had low initial aerobic fitness (measured as peak oxygen uptake during a graded exercise test, VO_2_peak) but had a 10% improvement in VO_2_peak following an incentivized, self-paced 16-week exercise program [15]. We also showed that several plasma amino acids and amino metabolites were altered with obesity [16]. The current analysis examines the effects of obesity and exercise training on circulating lipids in the same American Indian adolescents, which has not previously been reported in this underrepresented population. Our primary goal was to determine the magnitude of dyslipidemia with obesity and to assess whether improvements would result from exercise training. We measured standard lipids, oxLDL and oxHDL, and several individual fatty acids as part of a targeted lipidomics panel. We also compared the ratios of specific fatty acids that are surrogates of hepatic lipid metabolism and conducted correlational analysis to determine how specific lipid measurements varied with body composition and standard biochemical assessments.

## Methods

### Design

The results presented here are exploratory, secondary analyses from a randomized trial that was designed to test the effects of financial incentives on the frequency and duration of exercise performed by American Indian adolescents with obesity (Ob) [15]. The study was approved by the Institutional Review Boards of the Choctaw Nation of Oklahoma and the University of Oklahoma Health Sciences Center, respectively. The trial was registered at ClinicalTrials.gov (NCT01848353). Recruitment of participants began in June 2013. Primary data and sample collection was performed between June 2013 and January 2017. The main outcomes, including the CONSORT flow diagram, have been published [15].

### Participants

The study cohort was comprised of American Indian adolescents living in the Choctaw Nation Health Service Area of Southeast Oklahoma [15]. All participants were Tanner development stage ≥ 2, as determined by a pediatrician [17,18]. There were 74 participants with Ob in the current analyses, defined as having a body mass index (BMI) ≥ 95^th^ percentile for age and sex based on growth charts for children and adolescents from the United States Centers for Disease Control and Prevention [19]. The participants with Ob were 11.0 to 20.8 years old and had low habitual physical activity, defined as not being engaged in sports or exercise programs and attaining (via self-report) less than 30 minutes of structured moderate-to-vigorous intensity exercise on three or fewer days per week during the three months before enrollment. All the participants with Ob were assigned to the exercise intervention. For the current analysis, 52 of the participants with Ob had samples and data at baseline and the post-exercise period that could be used to evaluate the effects of exercise training. The control group with normal weight (NW) was comprised of 39 participants who had the same age range as the Ob group and a BMI between the 25th and 84th percentile for age and sex [19]. There was no criteria for history of physical activity for the NW group. The NW group completed all the same assessments as the Ob group upon enrollment but did not complete the exercise intervention. Participants from either group were excluded if they had confirmed diabetes or other potentially confounding health conditions, including active cardiovascular, endocrine or liver disease, pregnancy, polycystic ovary syndrome, kidney or other organ dysfunction, impaired physical mobility, anemia, symptoms of undiagnosed illness, regular tobacco use within the past 6 months, or substance abuse. We also excluded those who used medications to treat cardiometabolic conditions, promote weight loss, or that were known to influence lipid, glucose, or protein metabolism. Each participant, and the parents or guardians of participants under 18 years old, provided their informed, written consent and/or assent to enroll in the study in accordance with the Institutional Review Board guidelines. After enrollment, a clinician conducted a medical history and exam to confirm eligibility for the study. The final analytic sample of 74 Ob and 39 NW participants at baseline was reached after applying all exclusion criteria (S1 Fig). A total of 28 enrolled participants were excluded from the current analyses because they did not meet the inclusion criteria, voluntarily withdrew before completing baseline tests. Of the 74 members of the Ob group who completed baseline tests, 22 withdrew before completing follow-up testing after 16 weeks of exercise.

### Exercise training program

Upon completion of baseline physiological and biochemical testing, as described below, the Ob group were assigned to an exercise program that was 48-weeks in duration and subdivided into three, consecutive 16-week phases. Each phase was designed to test how different incentive structures would affect exercise frequency and/or duration, as previously described [15]. For the current analysis, we determined the effects of exercise training only in response to Phase 1 (weeks 1–16), and pooled data from all exercisers with available samples since there was no effect of the financial incentives on exercise frequency or duration during Phase 1.

The Ob group were instructed to exercise at their local wellness center operated by Choctaw Nation in Southeast Oklahoma [15]. Participants could exercise on schedules of their own choosing and could perform any type of aerobic or resistive exercise that was available at the center. Participants were instructed to complete at least three exercise sessions per week, with a minimum of 20 minutes per session of moderate-to-vigorous physical activity (MVPA), defined as activity that elicited a heart rate ≥ 70% of the peak heart rate recorded during a graded exercise test. During all exercise sessions, participants wore a chest strap heart rate monitor to record the duration and intensity of activity, the time spent in MVPA, and to provide feedback during and after exercise (Spirit System, Interactive Health Technologies, Austin, TX) [15].

### Physiological testing

All participants completed the following tests upon enrollment (referred to as baseline), and the Ob group repeated the same tests after 16 weeks of the exercise program. Body mass and height were used to calculate BMI (kg/m^2^), which was converted to BMI z-score using standard growth charts [19]. The BMI z-score expresses an participant’s BMI relative to the age- and sex-specific population mean, which is standardized using the United States Centers for Disease Control and Prevention 2000 growth charts [19]. A z-score of 0 represents the population average, while a z-score ≥ 1.65 corresponds approximately to the 95th percentile. Total body fat, fat-free mass, and trunk fat were measured using bioelectrical impedance (Model BC-418, Tanita Corporation, Arlington Heights, IL). A progressive intensity exercise test on stationary bicycle ergometer was used to measure peak values for heart rate and oxygen consumption rate (VO_2_peak) [15]. Daily ambulatory activity was measured with an accelerometer worn on the waist (Fitbit Zip, Fitbit Inc., San Francisco, CA) for seven days.

### Blood analyses

Venous blood was collected in the morning following an overnight fast. Glycated hemoglobin (HbA1c) was measured immediately on whole blood (Siemens DCA Vantage analyzer, Tarrytown, NY). After centrifugation, aliquots of plasma and serum were stored at −80°C until analysis. Plasma glucose was measured by the glucose oxidase method (2300STAT Plus, Yellow Springs Instruments, Yellow Springs, OH). Serum insulin was measured using a chemiluminescent enzyme-linked immunosorbent assay (ELISA, #80-INSHU-CH10, ALPCO, Salem, NH). Insulin resistance was calculated using glucose and insulin concentrations with the revised integrated homeostatic model of assessment (iHOMA2-IR) [20]. Serum total cholesterol, HDL-C, TG, and non-esterified fatty acids (NEFA) were measured using chromogenic assays (Wako Chemicals, Richmond, VA). LDL-C was calculated using a formula [21]. ELISAs were used to measure serum concentrations of oxHDL (MyBioSource, Inc., San Diego, CA), oxLDL (Mercodia, Uppsala, Sweden), C-reactive protein (Abcam, Cambridge, MA), and myeloperoxidase (R&D Systems, Minneapolis, MN).

A targeted approach was used to measure the plasma concentration of 11 individual fatty acids using ultra-performance liquid chromatography-mass spectrometry at the Mayo Clinic Metabolomics Core Laboratory (Rochester, MN) [22]. This analysis was performed in a subset of 26 girls and 30 boys from the Ob group and 15 girls and 20 boys in the NW group who had adequate samples available at the baseline assessment. Within the Ob group, 42 participants had plasma samples available for this analysis at baseline and after the 16-week exercise intervention. Samples were thawed on ice and spiked with ^13^C-labelled internal standards and 10-point standard curves were used to calculate the concentration of each fatty acid. A quality control sample and one of the concentration standards were injected throughout each sequence and used to calculate the intra- and inter-assay coefficients of variation, which averaged 2% and 9%, respectively, across analytes. The ratios of specific fatty acids were calculated to estimate the activities of the following intrahepatic enzymatic processes: stearoyl CoA desaturase 1 [ratio of palmitoleic (16:1)/palmitic (16:0), and oleic (18:1)/stearic (18:0) acids for conversion of 16- and 18-carbon fatty acids, respectively], fatty acid elongase [ratio of stearic (18:0)/palmitic (16:0), and oleic (18:1)/ palmitoleic (16:1) acids, for 16- and 18-carbon fatty acids, respectively], and delta 6 desaturase [ratio of linolenic (18:3)/linoleic (18:2) acids].

### Data analyses

All data analyses were performed, and figures were prepared using GraphPad Prism 10.5. Summary values for all variables are presented as mean ± standard deviation or median (interquartile range) for variables with equal or unequal variances, respectively. Between-group differences at baseline and within-group changes from the beginning to the end of the exercise program for the Ob group were evaluated with t-tests or Mann-Whitney tests, as appropriate. Nominal, uncorrected p-values are presented in figures and text since this was an exploratory secondary analysis. Power tests and *a priori* hypotheses were not established before initiating the lipid measurements. Nevertheless, for each cluster of related variables that were the focus of the current analysis (standard lipids, individual fatty acids, and fatty acid ratios used to estimate enzymatic activity, respectively) we also performed the two-stage linear step-up procedure developed by Benjamini, Krieger and Yekutieli, and as recommended in GraphPad Prism, to control for the false discovery rate [23]. The corrected p-value considered to be the threshold for a discovery (i.e., between group differences or within group change between time points) is reported for each cluster. Pearson’s or Spearman’s correlations were calculated for variables with equal or unequal variances, respectively, to determine strength of association among lipid analytes and physiological variables such as age, body fat, trunk fat, and insulin resistance. To determine the biological effect of sex, two-way analysis of variance tests were performed with the main factors of sex and study group. When significant interactions or main effects were identified, pairwise comparisons of means were performed with Fisher’s least significant difference tests that were not corrected for multiple comparisons. Statistical significance was set at P < 0.05.

## Results

### Baseline comparison between the NW and Ob groups

**Fig 1:**
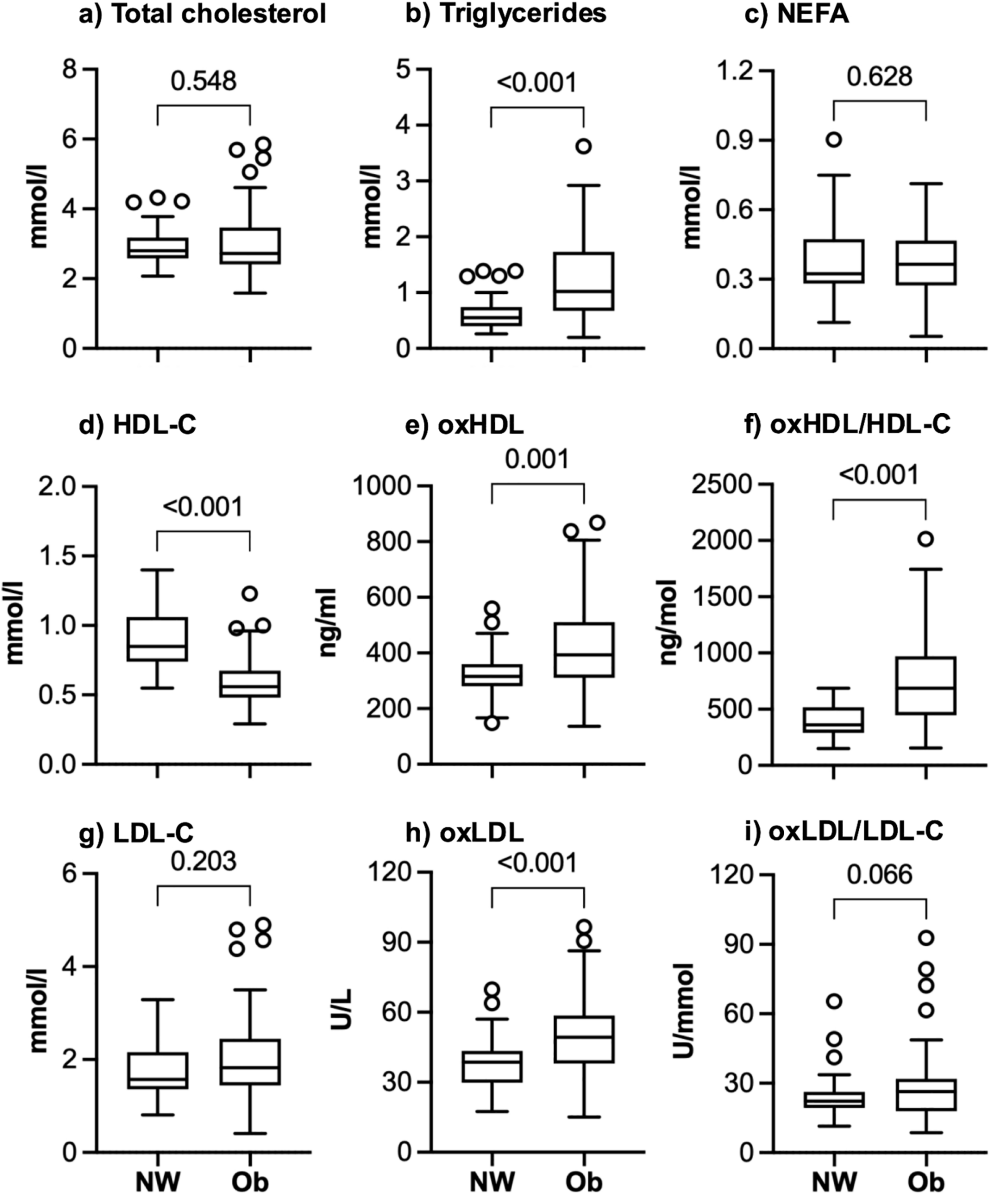
Concentrations of circulating lipids in adolescents with normal weight (NW) or obesity (Ob). Boxes show the median and interquartile range (IQR) for each group. Whiskers show the range of values within 1.5x of the IQR. Circles are outlier values outside of 1.5x of the IQR. Unadjusted P-values are shown for t-tests (NEFA, HDL-C) or Mann-Whitney tests (all other variables) for between group comparisons. A P-value < 0.071 is the threshold for false discovery rate after applying the two-stage linear step-up procedure of Benjamini, Krieger and Yekutieli for multiple comparisons. Results are for 39 NW and 74 Ob participants at the study baseline, before the Ob group started the exercise program.

As shown in Table 1, age, HbA1c, and myeloperoxidase were not different between the NW and Ob groups, but all other measures of body size and composition, aerobic fitness, physical activity, glucose, insulin, and insulin resistance differed significantly between groups. By design, at the time of enrollment none of the participants met the criteria for T2D according to guidelines from the American Diabetes Association [24], defined as having fasting glucose ≥ 7.0 mmol/l and/or HbA1c ≥ 6.5%. The Ob group had significantly higher values for TG (60% higher median for Ob), oxHDL (24%), and oxLDL (60%), and lower HDL-C (34%) than the NW group (Fig 1). Total cholesterol, NEFA, and LDL-C did not differ between the groups (Fig 1). The TG to HDL-C ratio, which was proposed as a surrogate marker of insulin resistance in children [25], was, median [interquartile range], 0.64 [0.42, 0.96] in the NW group and 1.55 [0.85, 2.16] in the Ob group (p < 0.001).

**Table 1:** Clinical and physiological characteristics (full cohort).

	NW	Ob	Difference (95% CI)	p-value
Age, y	14.5 ± 2.1	14.1 ± 2.2	−0.4 (−1.2, 0.5)	0.413
BMI, z-score	0.30 [−0.11, 0.78]	2.37 [2.15, 2.53]	2.07 (1.86, 2.27)	< 0.001
Fat-free mass, kg	40.7 [36.0, 48.8]	50.4 [45.0, 60.8]	9.7 (5.9, 13.6)	< 0.001
Body fat, kg	11.7 [8.4, 14.7]	39.3 [30.9, 51.9]	27.1 (23.1, 32.0)	< 0.001
Body fat, %	22 ± 7	43 ± 8	21 (18, 24)	< 0.001
Trunk fat, kg	4.9 [3.4, 6.5]	15.3 [13.0, 20.5]	10.5 (9.3, 12.7)	< 0.001
Trunk fat, %	17.4 ± 6.8	37.8 ± 7.0	20.4 (17.6, 23.2)	<0.001
VO_2_peak, ml/kg FFM/min	53.6 ± 10.6	34.5 ± 8.1	−19.2 (−22.8, −15.3)	< 0.001
Steps per day	8,899 ± 3,149	6,448 ± 2,901	−2,451 (−3,656, −1,247)	< 0.001
Glucose, mmol/l	4.97 [4.42, 5.14]	5.18 [4.92, 5.50]	0.21 (0.10, 0.39)	< 0.001
Insulin, pmol/l	33.6 [28.3, 42.8]	93.5 [60.0, 175.5]	59.9 (44.1, 80.8)	< 0.001
iHOMA2-IR	0.63 [0.53, 0.79]	1.76 [1.16, 3.26]	1.13 (0.80, 1.45)	< 0.001
HbA1c, %	5.4 ± 0.3	5.3 ± 0.3	−0.1 (−0.2, 0.1),	0.445
Myeloperoxidase, ng/ml	110 ± 103	97 ± 107	4 (−26, 8)	0.542
C-reactive protein, nmol/l	24.3 ± 29.3	96.7 ± 100.8	52.4 (22.0, 67.2)	< 0.001

Results for tests completed upon study enrollment (baseline) before the Ob group began the exercise intervention. Values presented as mean ± standard deviation for normally-distributed variables, or median [upper, lower interquartile range] for variables with unequal variances. Differences between groups are in absolute values with 95% confidence intervals (95% CI). Unadjusted P-values are for between group comparisons performed with unpaired t-tests or Mann-Whitney tests, as appropriate. BMI, body mass index; VO_2_peak, peak rate of oxygen uptake during cycling fitness test; FFM, fat-free mass; Steps per day, physical activity measured with accelerometer; iHOMA2-IR, interactive homeostasis model assessment 2, insulin resistance (unitless); HbA1c, glycated hemoglobin. The group with NW had 18 female and 21 male participants, while the group with Ob had 39 female and 35 male participants. For VO_2_peak 8 participants in the Ob group and 1 in the NW group had missing data. For step counts 4 participants in the Ob group and 2 in the NW group had missing data. Missing data were due to technical errors, incomplete data collection, or lost or broken equipment.

**Fig 2:**
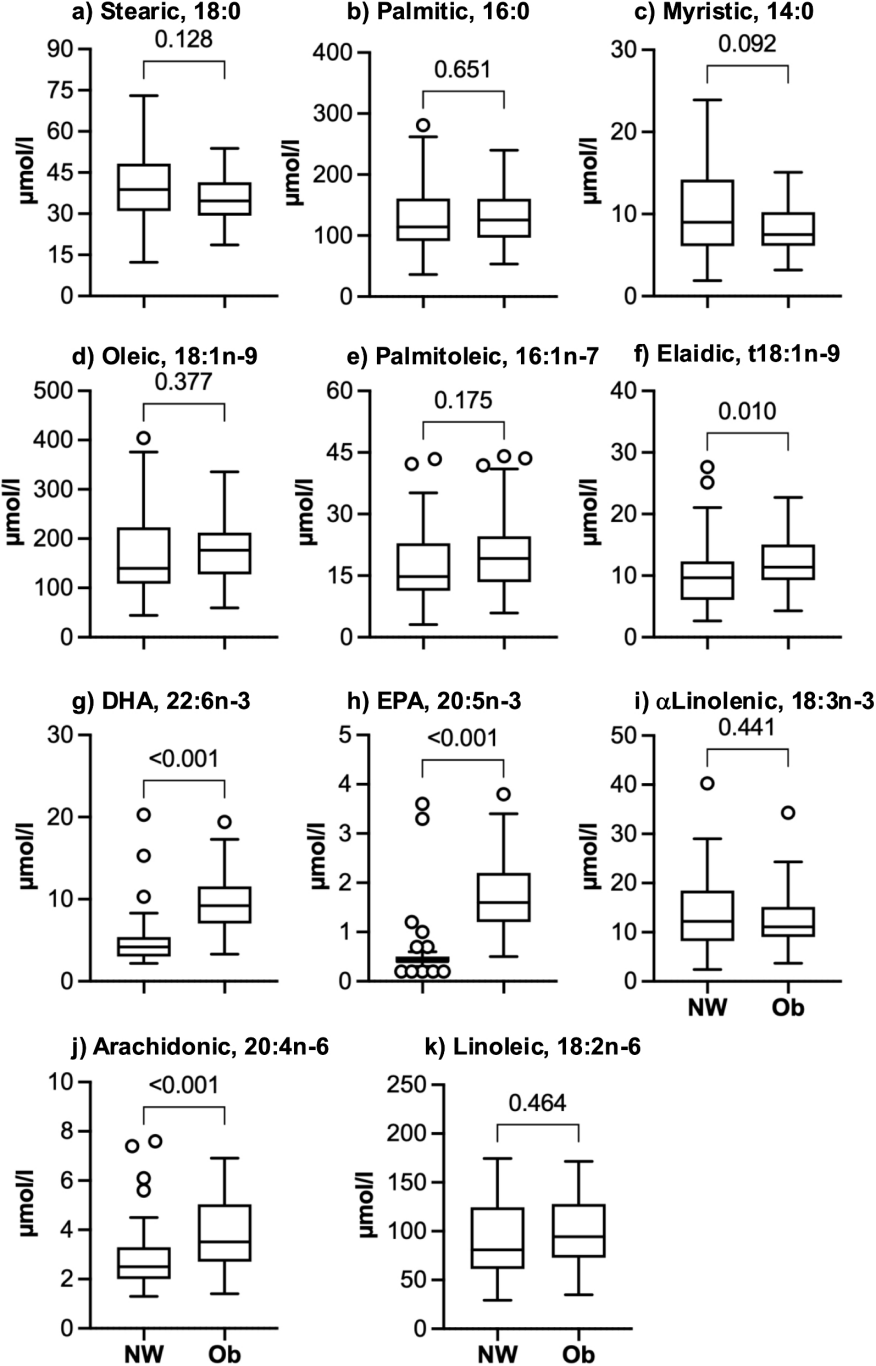
Concentrations of circulating fatty acids in adolescents with normal weight (NW) or obesity (Ob). Boxes show the median and interquartile range (IQR) for each group. Whiskers show the range of values within 1.5x of the IQR. Circles are outlier values outside of 1.5x of the IQR. Unadjusted P-values shown are for Mann-Whitney tests for between group comparisons. A P-value < 0.027 is the threshold for false discovery rate after applying the two-stage linear step-up procedure of Benjamini, Krieger and Yekutieli for multiple comparisons. Results are for 35 NW and 56 Ob participants at the study baseline, before the Ob group started the exercise program. EPA, eicosapentaenoic acid; DHA, docosahexaenoic acid.

**Fig 3:**
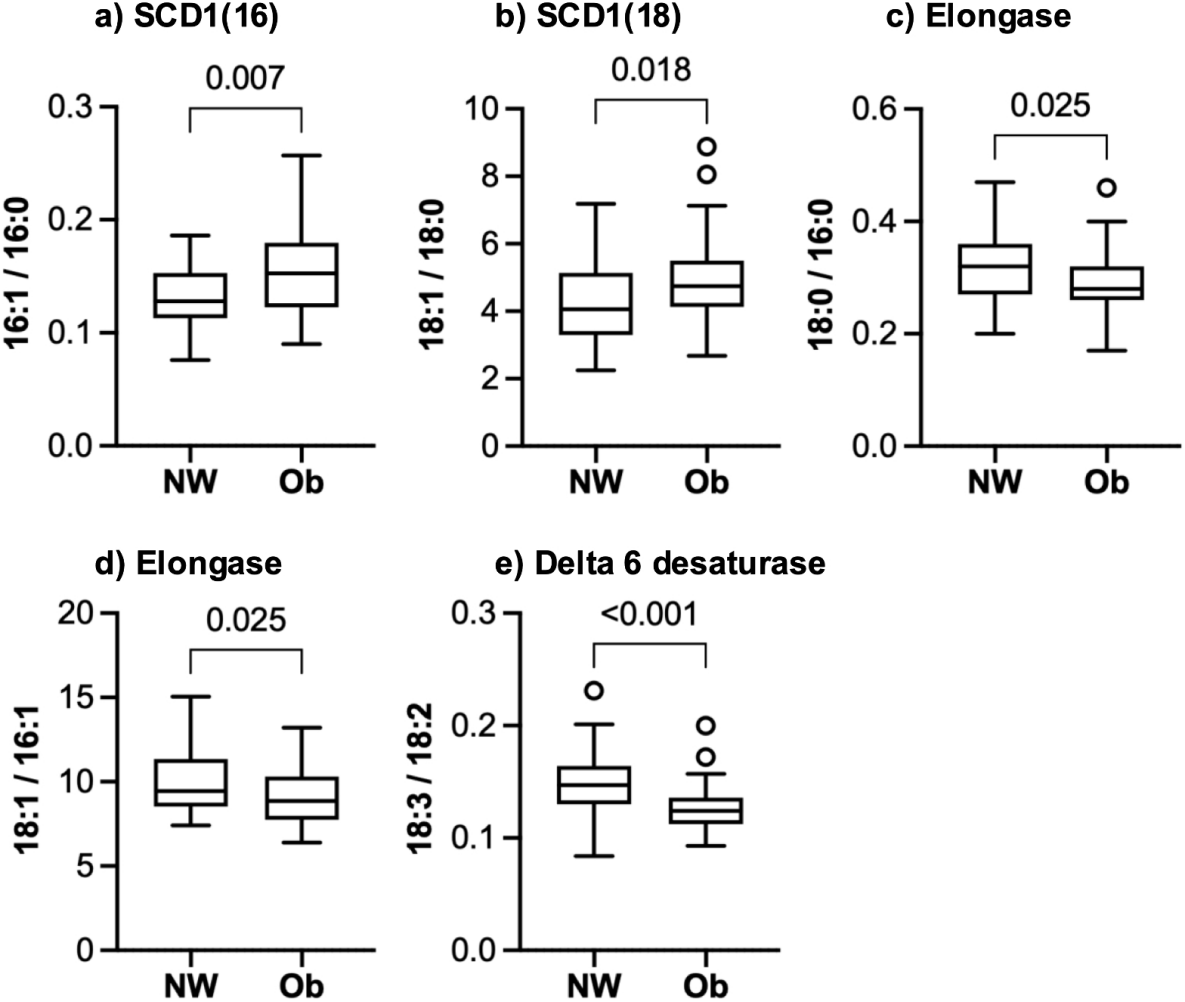
Estimated activities of stearoyl CoA desaturase 1 (SCD), elongase, and delta 6 desaturase. Boxes show the median and interquartile range (IQR) for each group. Whiskers show the range of values within 1.5x of the IQR. Circles are outlier values outside of 1.5x of the IQR. Unadjusted P-values are shown for Mann-Whitney tests for between group comparisons. After applying the two-stage linear step-up procedure of Benjamini, Krieger and Yekutieli for multiple comparisons, all adjusted P-values were < 0.026. Results are for 35 NW and 56 Ob participants at the study baseline, before the Ob group started the exercise program. Fatty acids used to calculate each enzymatic process are shown on the y-axes.

The subgroup of participants used for fatty acid analyses had physiological and clinical characteristics that were similar to the larger cohort, as shown in S1 Table. The Ob group had higher values for elaidic acid (19% higher median for Ob), docosahexaenoic acid (117%), eicosapentaenoic acid (272%), and arachidonic acid (38%), but stearic acid, palmitic acid, myristic acid, oleic acid, and palmitoleic acid did not differ between groups (Fig 2). As shown in Fig 3, estimates for stearoyl CoA desaturase were higher in the Ob than the NW group for both 16 and 18 carbon monounsaturated species (median values 19 and 17% higher, respectively). Elongase activity was 13% lower in the Ob group for 18:0/16:0 and 6% lower for 18:1/16:1. The estimate for delta 6 desaturase was also 16% lower in the Ob group.

To determine the biological impact of sex, we compared lipid values for boys and girls (S2–S4 Tables). There were sex differences in body composition and glucose concentration (higher in boys) within the NW group but not the Ob group. Insulin was higher in boys than girls in the Ob group only, and boys were more physically active than girls in both groups (S2 Table). However, the only sex difference in the lipid measurements, shown in S3 and S4 Tables, was lower TG in boys than girls in the NW group; all other comparisons were not statistically significant.

**Fig 4:**
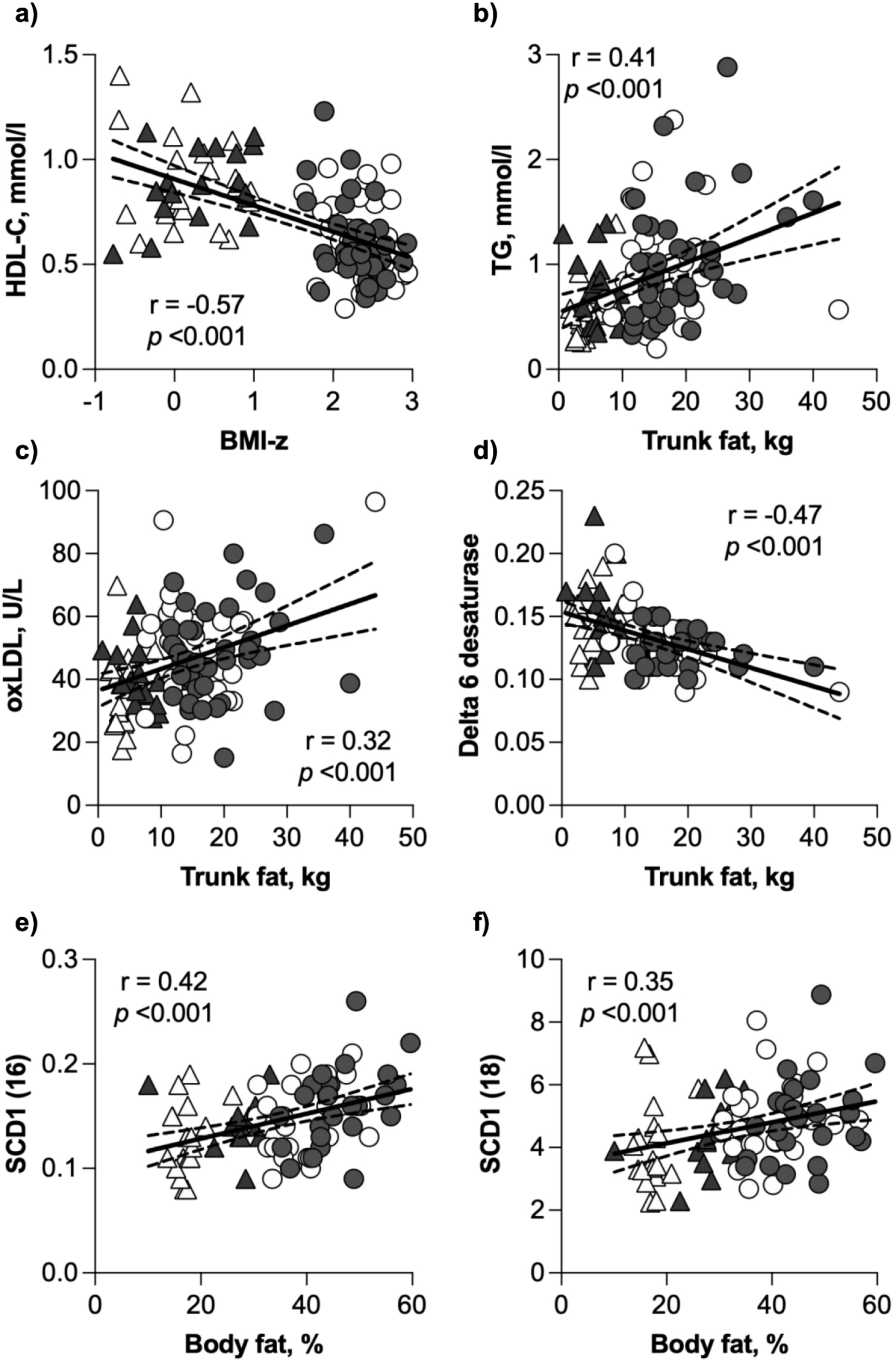
Correlations between selected lipids and indices of adiposity. TG, triglycerides; oxLDL, oxidized LDL; D6 ds, delta 6 desaturase; SCD1(16) and SCD1(18), stearoyl CoA desaturase 1 activity on 16- and 18-carbon monounsaturated fatty acids, respectively. Triangles and circles represent the NW and Ob groups, respectively. Closed symbols are girls, open symbols are boys, respectively. Regression lines (solid line), 95% confidence intervals (dashed lines), Spearman’s correlation coefficient (r), and p-values are for the line of best fit for the whole cohort.

Several of the lipids were associated with measures of adiposity. The correlations in Fig 4 were selected based on their clinical relevance and their significance in group comparisons; other lipid-anthropometric associations were explored but are not shown. HDL-C was inversely correlated with BMI z-score (Fig 4) in addition to total body fat and trunk fat, in kg, (Spearman’s r = −0.51 and −0.53, respectively, p < 0.001), total body fat and trunk fat, in % of mass (Pearson’s r = −0.57 and −0.49, respectively, p < 0.001), and iHOMA2-IR (Spearman’s r = −0.55, p < 0.001). TG, oxLDL and delta 6 desaturase were all positively correlated with trunk fat (Fig 4), while both the 16- and 18-carbon stearoyl-CoA desaturase 1 values were positively correlated with total body fat percentage (Fig 4). Like HDL-C, each of the other lipids in Fig 4 had similar, albeit smaller correlations with the other indices of adiposity. Additionally, TG concentration was positively correlated with insulin (r = 0.38), iHOMA2-IR (r = 0.38), oxHDL/HDL-C (r = 0.45), and oxLDL (r = 0.43; all (all Spearman’s, p < 0.01).

We explored whether there were associations between age or physical activity on the measurements. As expected with this age group, absolute values for fat mass and fat-free mass increased with age but body fat % (Spearman’s r = 0.043, p = 0.65) and trunk fat % (Spearman’s r = 0.112, p = 0.24) were not significantly correlated with age. None of the lipids shown in Fig 1 were significantly correlated with age, either within the whole group or within the NW or Ob groups when considered separately. For the FAs shown in Fig 2, there were significant inverse correlations with age, but only within the NW group for linolenic, linoleic, palmitoleic, oleic, elaidic, myristic, and palmitic acids (range of Pearson’s r values −0.38 to −0.46, p < 0.03). Although the NW and Ob groups differed in measured physical activity (steps per day shown in Table 1), there was considerable overlap with a range of 4,532–16,191 steps per day in the NW group and 2,224–18,041 steps per day in the Ob group. None of the lipid values were correlated with physical activity in the whole cohort or within the NW or Ob groups, except for a positive relationship between daily steps and HDL-C within the NW group (Pearson’s r = 0.38, p = 0.021).

### Changes in response to the exercise program

Of the 74 participants in the Ob group who completed baseline testing, 52 completed the exercise program and post-intervention tests (S1 Fig). There were no significant differences in any of the descriptive characteristics between those who completed the post-intervention tests versus those who did not. For example, baseline values for completers versus non-completers for several relevant variables differed by less than 6%: age (13.9 ± 2.1 versus 14.6 ± 2.4 years, p = 0.230), BMI z-score (2.31 ± 0.31 versus 2.41 ± 0.30, p = 0.199), body fat percentage (43.4 ± 7.8 versus 43.3 ± 7.2%, p = 0.955), VO_2_peak (34.5 ± 8.1 versus 34.8 ± 8.6 ml/kg FFM/min, p = 0.583), and physical activity (6,331 ± 3,054 versus 6,722 ± 2,556 steps per day, p = 0.583), respectively. Similarly, of the 52 participants who completed post-intervention testing, there were no differences in the descriptive characteristics between the 42 participants who had results for the fatty acid panel and the 10 who did not. Participants with versus without the fatty acid panel differed by ≤13% for age (13.8 ± 1.9. versus 14.1 ± 3.1 years, p = 0.805), BMI z-score (2.29 ± 0.33 versus 2.39 ± 0.19, p = 0.202), body fat percentage (43.4 ± 8.0 versus 43.3 ± 7.5%, p = 0.989), VO_2_peak (33.5 ± 7.2 versus 38.0 ± 10.1 ml/kg FFM/min, p = 0.220), and physical activity (6,369 ± 3,123 versus 6,179 ± 2,921 steps per day, p = 0.859), respectively.

Participants in the 16-week exercise program completed a median of 36 out of 48 expected exercise sessions with at least 20 minutes of MVPA (range: 7–71 sessions) and a median of 1 additional session that did not meet that threshold, with an average duration of 34 ± 8 minutes of MVPA per session. The cumulative exercise time per person over 16 weeks was 18.3 ± 8.6 hours (range: 11.4 to 22.6 hours). After 16 weeks, total body fat increased from baseline and trunk fat had a smaller, non-significant increase, but BMI-z and fat-free mass were not significantly changed (Table 2). There was a small but significant improvement in exercise capacity, shown by a 10% increase in peak oxygen consumption during the bicycle ergometer test. Physical activity, glucose, insulin, HbA1c, and the inflammatory markers, myeloperoxidase and C-reactive protein, were not significantly changed (Table 2). After the exercise program, there were no significant changes in most lipids measured. However, elaidic and myristic acids had statistically significant reductions, while palmitoleic and oleic acids and total NEFA had non-significant declines (Table 3).

**Table 2:** Clinical and physiological characteristics of the exercise group with obesity at baseline (Pre) and after the 16-week physical activity intervention (Post).

	Data pairs	Pre	Post	Change (95% CI)	p-value
BMI, z-score	52	2.31 ± 0.31	2.33 ± 0.31	0.02 (−0.01, 0.04)	0.167
Fat-free mass, kg	51	51.7 ± 10.7	52.3 ± 11.2	0.6 (−0.3, 1.5)	0.199
Body fat, kg	51	41.3 ± 15.8	43.1 ± 15.8	1.8 (0.7, 2.8)	0.001
Body fat, %	51	43.4 ± 7.8	44.3 ± 7.6	0.9 (0.1, 1.7)	0.027
Trunk fat, kg	49	16.5 ± 6.1	17.0 ± 5.7	0.5 (−0.1, 1.0)	0.070
Trunk fat, %	49	37.6 ± 7.1	38.7 ± 7.3	1.1 (−0.0, 2.3)	0.059
VO_2_peak, ml/kg FFM/min	43	33.8 ± 7.2	37.2 ± 8.3	3.3 (1.1, 5.6)	0.004
Steps per day	40	6,335 ± 3,223	6,181 ± 2,997	−154 (−1,171, 864)	0.762
Glucose, mmol/l	52	5.2 ± 0.4	5.6 ± 1.9	0.4 (−0.1, 0.9)	0.110
Insulin, pmol/l	52	133.5 ± 131.5	125.7 ± 84.7	−7.8 (−44.1, 28.9)	0.670
iHOMA2-IR	52	2.40 ± 2.17	2.45 ± 1.66	−0.04 (−0.59, 0.67)	0.891
HbA1c, %	52	5.3 ± 0.3	5.4 ± 0.6	0.1 (−0.0, 0.2)	0.114
Myeloperoxidase, ng/ml	51	93 ± 93	98 ± 75	5 (−18, 29)	0.647
C-reactive protein, nmol/l	51	83.6 ± 98.9	88.4 ± 89.4	4.7 (−21.9, 31.4)	0.722

Values shown as mean ± SD for the number of participants with complete pre and post intervention results shown in the second column. Changes from pre to post intervention are shown in absolute values with 95% confidence intervals (95% CI). Unadjusted P-values are shown for within-group comparisons performed with paired t-tests for all variables. Results are presented only for participants with both baseline and post-intervention values for a given variable. Missing data for body composition, aerobic capacity, and physical activity were attributable to technical errors, incomplete data collection, or lost or broken equipment. BMI, body mass index; VO_2_peak, peak rate of oxygen uptake during cycling fitness test; FFM, fat-free mass; Steps per day, physical activity measured with accelerometer; iHOMA2-IR, interactive homeostasis model assessment 2, insulin resistance (unitless); HbA1c, glycated hemoglobin.

**Table 3:** Circulating lipids in the group with obesity at baseline (Pre) and after the 16-week physical activity intervention (Post).

	Pre	Post	Change (95% CI)	p-value
Total cholesterol, mmol/l	2.96 ± 0.92	3.11 ± 0.99	0.15 (−0.06, 0.36)	0.150
HDL-C, mmol/l	0.61 ± 0.17	0.59 ± 0.14	−0.02 (−0.05, 0.01)	0.257
Triglycerides, mmol/l	0.96 ± 0.53	1.09 ± 0.73	0.13 (−0.06, 0.32)	0.165
LDL-C, mmol/l	1.99 ± 0.95	2.14 ± 0.93	0.15 (−0.06, 0.35)	0.168
NEFA, mmol/l	0.389 ± 0.157	0.346 ± 0.188	−0.045 (−0.096, 0.007)	0.087
oxLDL, U/L	49.5 ± 14.6	52.3 ± 18.3	2.8 (−2.3, 8.0)	0.271
oxLDL/LDL-C, U/mmol	28.5 ± 15.7	27.7 ± 11.3	−0.8 (−5.8, 4.2)	0.746
oxHDL, ng/ml	417 ± 180	418 ± 177	1 (−46, 47)	0.979
oxHDL/HDL-C, ng/mol	743 ± 390	754 ± 373	11 (−82, 105)	0.806
Stearic acid (18:0), µmol/l	35.07 ± 9.08	32.95 ± 10.89	−2.12 (−5.19, 0.94)	0.170
Palmitic acid (16:0), µmol/l	127.27 ± 44.00	116.47 ± 50.79	−10.81 (−23.68, 2.06)	0.097
Myristic acid (14:0), µmol/l	8.12 ± 3.08	7.12 ± 3.57	−1.00 (−1.93, −0.07)	0.036
Oleic acid (18:1), µmol/l	172.44 ± 66.95	153.93 ± 69.07	−18.51 (−37.33, 0.32)	0.054
Palmitoleic acid (16:1), µmol/l	20.46 ± 9.98	17.90 ± 10.07	−2.56 (−5.38, 0.26)	0.074
Elaidic acid (t18:1), µmol/l	11.95 ± 4.85	10.32 ± 4.75	−1.63 (−3.20, −0.52)	0.043
Docosahexaenoic acid (22:6), µmol/l	9.31 ± 3.16	8.66 ± 3.13	−0.65 (−1.60, 0.29)	0.169
Eicosapentaenoic acid (20:5), µmol/l	1.92 ± 1.32	1.97 ± 1.64	0.05 (−0.23, 0.33)	0.703
Linoleic acid (18:3), µmol/l	12.58 ± 5.7	11.45 ± 5.94	−1.13 (−3.16, 0.89)	0.265
Arachidonic acid (20:4), µmol/l	3.78 ± 1.25	3.55 ± 1.31	−0.23 (−0.69, 0.23)	0.317
Linoleic acid (18:2), µmol/l	97.91 ± 34.45	88.73 ± 40.17	−9.18 (−20.85, 2.49)	0.120
SCD1(16:1/16:0)	0.16 ± 0.04	0.15 ± 0.03	−0.01 (−0.02, 0.00)	0.117
SCD1(18:1/18:0)	4.83 ± 1.24	4.61 ± 1.35	−0.22 (−0.62, 0.19)	0.285
Elongase (18:0/16:0)	0.29 ± 0.05	0.30 ± 0.07	0.01 (−0.01, 0.03)	0.152
Elongase (18:1/16:1)	8.89 ± 1.49	9.24 ± 1.76	0.35 (−0.20, 0.89)	0.204
Delta 6 desaturase (18:3/18:2)	0.13 ± 0.02	0.13 ± 0.02	0.00 (−0.01, 0.01)	0.814

Values shown as mean ± SD. Unadjusted P-values are shown for within-group comparisons performed with paired t-tests for all variables. After applying the two-stage linear step-up procedure of Benjamini, Krieger and Yekutieli for multiple comparisons, all adjusted P-values were > 0.343. For the standard and oxidized lipids (cholesterol, HDL-C, triglycerides, LDL-C, oxHDL, oxLDL) the sample size was 52. For the individual fatty acids, the sample size was 42, for all but elaidic acid (n = 41).

Since there was a broad range of exercise sessions performed, we explored whether any of the outcome variables were related to the frequency or volume of exercise completed. The change in VO_2_peak was positively correlated with the number of exercise sessions performed (Pearson’s r = 0.37, p = 0.017) and the total hours of exercise (Pearson’s r = 0.40, p = 0.010). However, exercise behavior (number of exercise sessions, time spent exercising) was not significantly correlated with changes in any of the circulating lipids measured in this study.

## Discussion

In this exploratory analysis, we found that the concentrations of several plasma lipids were altered in American Indian adolescents with obesity but there were only minimal changes in response to an incentivized 16-week exercise program. Despite having fasting glucose and HbA1c within the normal range, the Ob group had several known risk factors for future development of T2D, including elevated body fat and insulin resistance, and lower physical activity and aerobic fitness compared to the NW group. The lower HDL-C and higher TG in the Ob group agrees with the prior studies demonstrating altered lipid profiles in adolescents with obesity [10]. The positive correlation between TG and insulin resistance is consistent with reports of lipid accumulation promoting steatotic liver disease and insulin resistance through decreased expression of Kruppel-like transcription factor 16 [3]. Fasting TG is a useful measurement for identifying insulin resistance in apparently healthy people [26]. Additionally, we found a negative correlation between HDL-C and insulin resistance. It was previously shown that insulin resistance leads to low HDL-C levels by enhancing CETP-mediated exchange of TG and cholesterol ester between HDL and triglyceride-rich lipoproteins [27]. Furthermore, lipolytic action of hepatic lipase in insulin resistance lowers HDL-C [27].

While prior studies have shown that American Indian adolescents with obesity have elevated cardiometabolic disease risk, including adverse lipid profiles and insulin resistance [28,29], we are not aware of studies that performed detailed lipid analysis in this population. Wheelock, et al. [28] reported that BMI percentile was positively correlated with TG and inversely correlated with HDL-C in a large cohort of American Indian children and adolescents who were studied in Arizona over four decades [28]. More recently, results from the Strong Heart Family Study showed that American Indians who were 15–19 years old and living in Arizona, Oklahoma, or the Dakotas, had a dyslipidemia prevalence of 55%, with an even higher prevalence of 78% in adults 30–39 years old [29]. Those reports underline the potentially high risk of cardiometabolic disease in this population. Our study is among the first to extend those findings by reporting values for oxidized lipids (oxHDL, oxLDL), targeted plasma fatty acid profiling, and enzymatic activity estimates (e.g., SCD1, elongase) in American Indian adolescents. These advanced lipidomic assessments provide a more nuanced picture of lipid metabolism and potential mechanisms linking obesity to cardiometabolic risk in this group.

A novel finding was that oxHDL and oxLDL were increased in the Ob group, and there were no differences between boys and girls in either the NW or Ob group. Previously, we reported that oxLDL and oxHDL were increased in adolescents with obesity and T2D, particularly boys, but not with obesity alone [11]. The increased concentration of these oxidized lipid particles suggests that the adolescents with obesity in the current study are at a higher risk for developing cardiometabolic disorders. This risk is further demonstrated by the fact that there were 3 participants in the Ob group who developed T2D within 16–20 weeks after enrollment, as previously described [15], although each of those participants withdrew from the study and their results were not included in this analysis. In adults, oxLDL has been shown to be pro-atherosclerotic [30]. The finding of lower HDL-C concentration in the Ob group suggests that there is lower capacity for reverse cholesterol transport, which could be further exacerbated by oxidative damage to HDL [31]. HDL also has a role in protecting LDL from oxidation, so the increased oxHDL may contribute to the increase in oxLDL. We expected to find that myeloperoxidase was increased in the Ob group since it is produced by macrophages and promotes the formation of oxHDL and oxLDL [31,32]. While myeloperoxidase did not differ between groups, the Ob group had higher C-reactive protein and higher insulin resistance, demonstrating further signs of hepatic inflammation and metabolic stress that contribute to cardiovascular disease risk. Likewise, estimates for SCD1 activity were increased, and elongase and delta 6 desaturase activity were decreased in the Ob group, which collectively suggest altered hepatic lipid metabolism. Similar differences in the fatty acid ratios used to calculate these lipid metabolism activities have been reported in children and adults with insulin resistance, steatotic liver disease, and T2D [33–36], which further supports the increased cardiometabolic risk in the current group of Ob adolescents.

The Ob group had a higher plasma concentration of elaidic acid, a trans fatty acid. Consumption of trans fatty acids may cause weight gain and insulin resistance through reduction of muscle Akt phosphorylation [37]. In monkeys, trans fatty acids increase intra-abdominal deposition of fat, even without excessive calorie intake [38]. Recent evidence from mice and humans showed that elaidic acid can also be produced by a strain of gut bacteria that are found in higher abundance in those with obesity or T2D [39] so it is unclear how much of the difference in plasma elaidic acid was due to dietary intake versus endogenous production. The blood samples in this study were collected before January 2017. The United States Food and Drug Administration’s ban on trans fats in the food supply began in 2015 but did not take full effect until June 2018, so it is likely that the elaidic acid values reported are at least partly reflective of dietary intake.

There was a positive correlation between SCD1(16) and body fat. This is consistent with a study that demonstrated that palmitoleic acid was positively related to abdominal obesity in children, possibly because the activation of SCD1 is not sufficiently suppressed by leptin [40]. High SCD1 activity has previously been associated with insulin resistance in adults with obesity [41]. SCD1 is also considered to be a potential drug target for management of obesity and diabetes because SCD1 inhibition decreases lipogenesis and increases GLUT-4 mediated glucose uptake in skeletal muscle [42]. Surprisingly, we found EPA and DHA were increased in the Ob group, which contradicts prior studies demonstrating a role of omega-3 fatty acids in reducing adipose tissue inflammation and therefore the risk of obesity and cardiometabolic syndrome [43]. However, arachidonic acid was increased in the Ob group, which aligns with research showing that increased omega-6 fatty acids are associated with obesity [44].

We expected that exercise training would partially correct some of the plasma lipid concentrations that were altered in the Ob group, even in the absence of a change in BMI. Although aerobic fitness increased in response to training, the lack of significant changes in more than two individual fatty acids demonstrates that the volume and/or type of exercise performed was insufficient for improvement in those metabolic pathways in this group of adolescents. Participants were given the flexibility to exercise on their own schedule, but compliance with the exercise target was lower than expected, with an average of only two sessions per week. Barriers to compliance included lack of transportation and competing time demands [15]. When previously inactive children with excess weight participated in a highly structured 8-month aerobic exercise program that was performed every weekday as part of an after school program, there were large improvements in aerobic fitness and HDL-C [45]. This suggests that a longer, more frequent exercise intervention is probably required to produce a clear clinical benefit in plasma lipids in children and adolescents with excess weight. A systematic review showed that aerobic exercise for 60 minutes on three or more days per week at moderate intensity can result in lowered LDL-C and TG concentrations in children with obesity [46]. That review also showed that higher intensity exercise is beneficial for increasing HDL-C concentration. Another study showed that LDL-C may decline in children who reduce dietary intake of high-fat vegetable oils as part of a two-year lifestyle intervention that included a physical activity component [47]. Thus, in addition to exercise, dietary changes may be necessary to significantly change plasma lipids in this age group.

A strength of the current study is the demonstration of dyslipidemia and increased cardiometabolic disease risk in American Indian adolescents who have excess body weight and lower physical activity and aerobic fitness than their peers with normal weight. This is a group of people that are often underrepresented in clinical research despite having high rates of T2D [14]. A limitation of our investigation was that diet was not controlled prior to the study visits and dietary history was not assessed. That information could have been helpful for understanding the patterns of lipids observed. Differences among participants in their diet, particularly fat intake, could have added variability to the plasma lipid outcomes or influenced the results in ways that were not measured. However, fasting concentrations of lipids are also determined by the combined actions of lipolysis, tissue uptake, and multiple metabolic processes in the liver and other organs, so dietary composition is only a piece of the overall picture. Another potential limitation is that blood sampling was only performed in the fasting state so additional differences in postprandial lipids may exist and could contribute to overall health status. An aspect of the study design that may have influenced the comparisons between the NW and Ob groups was that we did not have a criteria for history of physical activity for the NW group. The NW group had significantly higher daily step counts than the Ob group at baseline. Although the daily step count during the observation period was not correlated with any of the lipid measurements, the overall difference in habitual physical activity may have influenced lipid values and represents a potential source of bias in the between-group comparisons. Future studies that match groups on physical activity level in addition to body weight status would help clarify these relationships. Finally, since this was an exploratory analysis without an *a priori* power calculation, the sample size may not have been adequate to detect some potential differences between groups or in response to the intervention.

In conclusion, several plasma lipids were altered in American Indian adolescents with excess weight, including TG, HDL-C, oxHDL, oxLDL, and individual fatty acids. However, total cholesterol, LDL-C, and total NEFA did not differ. In response to the exercise program, there was a small increase in fitness, but only minor reductions in fatty acids, underscoring the need for more effective and sustainable lifestyle interventions that can reduce cardiometabolic disease risk in this population.

## Supporting information

S1 Table Characteristics of participant subset used for fatty acid panel. Results are from tests completed upon study enrollment (baseline) before the Ob group began the exercise intervention. Values presented as mean ± standard deviation for normally-distributed variables, or median [upper, lower interquartile range] for variables with unequal variances. Differences between groups are in absolute values with 95% confidence intervals (95% CI). P-values are for between group comparisons performed with unpaired t-tests or Mann-Whitney tests, respectively. BMI, body mass index; VO_2_peak, peak rate of oxygen uptake during cycling fitness test; FFM, fat-free mass; Steps per day, physical activity measured with accelerometer; iHOMA2-IR, interactive homeostasis model assessment 2, insulin resistance (unitless); HbA1c, glycated hemoglobin. The group with normal weight (NW) had 15 female and 20 male participants; the group with obesity (Ob) had 26 female and 30 male participants.
(PDF)

S2 Table Clinical and physiological characteristics for females and males within each study group. Results are from tests completed upon study enrollment (baseline) before the Ob group began the exercise intervention. Values presented as mean ± standard deviation. P-values are from a two-way ANOVA with sex and study group as the factors. Individual means were compared with Fisher’s least significant difference tests uncorrected for multiple testing. * different from females within group, p < 0.05; † different from NW group of the same sex, p < 0.05. BMI, body mass index; VO_2_peak, peak rate of oxygen uptake during cycling fitness test; FFM, fat-free mass; iHOMA2-IR, interactive homeostasis model assessment 2, insulin resistance; HbA1c, glycated hemoglobin. The group with normal weight had 18 female and 21 male participants, while the group with obesity had 39 female and 35 male participants.
(PDF)

S3 Table Standard lipid values for females and males within each study group. Results are from tests completed upon study enrollment (baseline) before the Ob group began the exercise intervention. Values presented as mean ± standard deviation. P-values are from a two-way ANOVA with sex and study group as the factors. Individual means were compared with Fisher’s least significant difference tests uncorrected for multiple testing. NEFA, non-esterified fatty acids. * different from females within group, p < 0.05; † different from NW group of the same sex, p < 0.05. The group with normal weight had 18 female and 21 male participants, while the group with obesity had 39 female and 35 male participants.
(PDF)

S4 Table Fatty acids and estimates of lipid enzymes for females and males within each study group. Results are from tests completed upon study enrollment (baseline) before the Ob group began the exercise intervention. All values presented as mean ± standard deviation. Individual fatty acids are presented as µmol/l. Enzyme estimates are presented as ratios of fatty acids and are unitless. P-values are from a two-way ANOVA with sex and study group as the factors. Individual means were compared with Fisher’s least significant difference tests uncorrected for multiple testing. SCD1, stearoyl CoA desaturase 1. * different from females within group, p < 0.05; † different from NW group of the same sex, p < 0.05. The group with normal weight had 15 female and 20 male participants, while the group with obesity had 26 female and 30 male participants.
(PDF)

S1 Fig Flow diagram showing the number of participants enrolled into the study and the numbers used for analyses. Due to sample availability, the panel of individual fatty acids was performed on a subset of participants who completed primary tests at baseline at after the 16-week exercise program.
(TIF)
