# Supplementary material for: Effect of obesity and exercise training on circulating lipids in American Indian adolescents
Source: PLoS One. 2025 Dec 16;20(12):e0338547. doi: 10.1371/journal.pone.0338547 (PMC12707644; doi:10.1371/journal.pone.0338547)
Supplement: S1 Table — Results are from tests completed upon study enrollment (baseline) before the Ob group began the exercise intervention. Values presented as mean ± standard deviation for normally-distributed variables, or median [upper, lower interquartile range] for variables with unequal variances. Differences between groups are in absolute values with 95% confidence intervals (95% CI). P-values are for between group comparisons performed with unpaired t-tests or Mann-Whitney tests, respectively. BMI, body mass index; VO2peak, peak rate of oxygen uptake during cycling fitness test; FFM, fat-free mass; Steps per day, physical activity measured with accelerometer; iHOMA2-IR, interactive homeostasis model assessment 2, insulin resistance (unitless); HbA1c, glycated hemoglobin. The group with normal weight (NW) had 15 female and 20 male participants; the group with obesity (Ob) had 26 female and 30 male participants. (PDF) [file pone.0338547.s001.pdf]

S1 Table. Characteristics of participant subset used for fatty acid panel.

	NW	Ob	Difference (95% CI)	p-value
Age, y	14.3 ± 1.7	13.9 ± 1.7	-0.4 (-1.1, 0.4)	0.305
BMI, z-score	0.31 [-0.03, 0.78]	2.39 [2.15, 2.53]	2.08 (1.80, 2.22)	< 0.001
Fat-free mass, kg	40.7 [36.0, 48.8]	50.4 [45.0, 61.5]	9.7 (5.5, 14.5)	< 0.001
Body fat, kg	11.7 [9.2, 14.6]	39.2 [29.9, 53.2]	27.5 (22.3, 32.7)	< 0.001
Body fat, %	22 ± 7	43 ± 6	21 (18, 24)	< 0.001
Trunk fat, kg	4.9 [3.4, 6.5]	15.4 [12.3, 20.2]	10.5 (9.2, 12.9)	< 0.001
Trunk fat, %	17.0 ± 6.4	37.7 ± 7.0	20.7 (17.8, 23.6)	<0.001
VO_2peak, ml/kg FFM/min	54.6 ± 10.6	33.2 ± 8.2	-21.4 (-25.5, -17.4)	< 0.001
Steps per day	9,202 ± 3,157	6,159 ± 2,908	-3,043 (-4,363, -1,723)	< 0.001
Glucose, mmol/l	4.94 ± 0.30	5.18 ± 0.35	0.24 (0.10, 0.38)	< 0.001
Insulin, pmol/l	33.0 [28.3, 40.4]	93.5 [63.0, 170.3]	60.5 (41.7, 81.2)	< 0.001
iHOMA2-IR	0.62 [0.53, 0.76]	1.76 [1.20, 3.16]	1.14 (0.77, 1.45)	< 0.001
HbA1c, %	5.4 ± 0.3	5.3 ± 0.3	-0.1 (-0.2, 0.1),	0.307
Myeloperoxidase, ng/ml	73 [48, 124]	76 [39, 117]	3 (-29, 11)	0.358
C-reactive protein, nmol/l	11.3 [3.4, 30.3]	58.7 [20.4, 107.5]	47.4 (16.5, 56.7)	< 0.001

Results are from tests completed upon study enrollment (baseline) before the Ob group began the exercise intervention. Values presented as mean ± standard deviation for normally-distributed variables, or median [upper, lower interquartile range] for variables with unequal variances. Differences between groups are in absolute values with 95% confidence intervals (95% CI). P-values are for between group comparisons performed with unpaired t-tests or Mann-Whitney tests, respectively. BMI, body mass index; VO_2peak, peak rate of oxygen uptake during cycling fitness test; FFM, fat-free mass; Steps per day, physical activity measured with accelerometer; iHOMA2-IR, interactive homeostasis model assessment 2 for insulin resistance (unitless); HbA1c, glycated hemoglobin. The group with normal weight (NW) had 15 female and 20 male participants; the group with obesity (Ob) had 26 female and 30 male participants.
