# Supplementary material for: Effect of obesity and exercise training on circulating lipids in American Indian adolescents
Source: PLoS One. 2025 Dec 16;20(12):e0338547. doi: 10.1371/journal.pone.0338547 (PMC12707644; doi:10.1371/journal.pone.0338547)
Supplement: S2 Table — Results are from tests completed upon study enrollment (baseline) before the Ob group began the exercise intervention. Values presented as mean ± standard deviation. P-values are from a two-way ANOVA with sex and study group as the factors. Individual means were compared with Fisher’s least significant difference tests uncorrected for multiple testing. * different from females within group, p < 0.05; † different from NW group of the same sex, p < 0.05. BMI, body mass index; VO2peak, peak rate of oxygen uptake during cycling fitness test; FFM, fat-free mass; iHOMA2-IR, interactive homeostasis model assessment 2, insulin resistance; HbA1c, glycated hemoglobin. The group with normal weight had 18 female and 21 male participants, while the group with obesity had 39 female and 35 male participants. (PDF) [file pone.0338547.s002.pdf]

S2 Table. Clinical and physiological characteristics for females and males within each study group.

	NW Females	NW Males	Ob Females	Ob Males	P-value for sex	P-value for group	P-value for interaction
Age, y	14.4 ± 2.1	14.5 ± 2.1	14.7 ± 2.4	13.4 ± 1.8	0.117	0.357	0.080
BMI, z-score	0.33 ± 0.56	0.20 ± 0.52	2.32 ± 0.29†	2.36 ± 0.33†	0.578	<0.001	0.288
Fat-free mass, kg	38.1 ± 4.8	46.6 ± 8.7*	51.5 ± 6.9†	55.9 ± 16.3†	0.004	<0.001	0.342
Body fat, kg	15.2 ± 5.0	9.7 ± 2.7	46.3 ± 16.2†	38.8 ± 15.7*†	0.014	<0.001	0.656
Body fat, %	28 ± 6	17 ± 3*	46 ± 7†	40 ± 7*†	<0.001	<0.001	0.065
Trunk fat, kg	6.6 ± 2.8	4.1 ± 1.7	19.2 ± 6.7†	15.6 ± 6.5*†	<0.001	0.007	0.606
Trunk fat, %	22 ± 7	13 ± 4*	40 ± 7†	35 ± 6*†	<0.001	<0.001	0.156
VO_2peak, ml/kg FFM/min	48.9 ± 8.3	57.6 ± 10.9*	32.5 ± 6.1†	36.6 ± 9.6†	<0.001	<0.001	0.201
Steps per day	7,718 ± 2,836	9,904 ± 3,116*	5,707 ± 2,368†	7,278 ± 3,239*†	0.002	<0.001	0.600
Glucose, mmol/l	4.80 ± 0.25	5.08 ± 0.27*	5.20 ± 0.45†	5.20 ± 0.38	0.072	<0.001	0.046
Insulin, pmol/l	39.5 ± 11.7	33.6 ± 17.6	194.5 ± 220.5†	103.0 ± 75.4*	0.075	<0.001	0.117
iHOMA2-IR	0.73 ± 0.22	0.66 ± 0.30	3.40 ± 3.43†	1.90 ± 1.33*†	0.069	<0.001	0.99
HbA1c, %	5.4 ± 0.4	5.4 ± 0.2	5.3 ± 0.3	5.4 ± 0.3	0.307	0.485	0.210

Results are from tests completed upon study enrollment (baseline) before the Ob group began the exercise intervention. Values presented as mean ± standard deviation. P-values are from a two-way ANOVA with sex and study group as the factors. Individual means were compared with Fisher's least significant difference tests uncorrected for multiple comparisons. * different from females within group, $p < 0.05$; † different from NW group of the same sex, $p < 0.05$. BMI, body mass index; VO_2peak, peak rate of oxygen uptake during cycling fitness test; FFM, fat-free mass; iHOMA2-IR, interactive homeostasis model assessment 2 for insulin resistance; HbA1c, glycated hemoglobin. The group with normal weight had 18 female and 21 male participants, while the group with obesity had 39 female and 35 male participants.
