# Supplementary material for: Effect of obesity and exercise training on circulating lipids in American Indian adolescents
Source: PLoS One. 2025 Dec 16;20(12):e0338547. doi: 10.1371/journal.pone.0338547 (PMC12707644; doi:10.1371/journal.pone.0338547)
Supplement: S3 Table — Results are from tests completed upon study enrollment (baseline) before the Ob group began the exercise intervention. Values presented as mean ± standard deviation. P-values are from a two-way ANOVA with sex and study group as the factors. Individual means were compared with Fisher’s least significant difference tests uncorrected for multiple testing. NEFA, non-esterified fatty acids. * different from females within group, p < 0.05; † different from NW group of the same sex, p < 0.05. The group with normal weight had 18 female and 21 male participants, while the group with obesity had 39 female and 35 male participants. (PDF) [file pone.0338547.s003.pdf]

S3 Table. Standard lipid values for females and males within each study group.

	NW Females	NW Males	Ob Females	Ob Males	P-value for sex	P-value for group	P-value for interaction
Total cholesterol, mmol/l	2.96 ± 0.50	2.91 ± 0.63	2.89 ± 0.92	3.05 ± 0.90	0.748	0.830	0.504
HDL-C, mmol/l	0.88 ± 0.18	0.90 ± 0.23	0.60 ± 0.19†	0.62 ± 0.18†	0.625	<0.001	0.942
Triglycerides, mmol/l	0.75 ± 0.33	0.52 ± 0.26*	1.02 ± 0.55†	0.93 ± 0.50†	0.079	<0.001	0.476
LDL-C, mmol	1.78 ± 0.56	1.76 ± 0.60	1.91 ± 0.90	2.09 ± 0.95	0.635	0.152	0.545
NEFA, mmol/l	0.408 ± 0.159	0.373 ± 0.190	0.347 ± 0.129	0.405 ± 0.153	0.701	0.633	0.134
oxLDL, U/L	40.7 ± 9.5	36.7 ± 11.9	48.5 ± 15.1	50.0 ± 17.6†	0.658	<0.001	0.363
oxLDL/LDL-C, U/mmol	24.6 ± 8.6	22.8 ± 11.5	29.7 ± 17.3	26.5 ± 12.6	0.368	0.113	0.790
oxHDL, ng/ml	330 ± 94	321 ± 83	424 ± 138†	417 ± 192†	0.769	0.001	0.958
oxHDL/HDL-C, ng/mol	397 ± 145	382 ± 141	757 ± 297†	753 ± 436†	0.869	<0.001	0.930

Results are from tests completed upon study enrollment (baseline) before the Ob group began the exercise intervention. Values presented as mean ± standard deviation. P-values are from a two-way ANOVA with sex and study group as the factors. Individual means were compared with Fisher's least significant difference tests uncorrected for multiple comparisons. NEFA, non-esterified fatty acids. * different from females within group, $p < 0.05$; † different from NW group of the same sex, $p < 0.05$. The group with normal weight had 18 female and 21 male participants, while the group with obesity had 39 female and 35 male participants.
