# Supplementary material for: Effect of obesity and exercise training on circulating lipids in American Indian adolescents
Source: PLoS One. 2025 Dec 16;20(12):e0338547. doi: 10.1371/journal.pone.0338547 (PMC12707644; doi:10.1371/journal.pone.0338547)
Supplement: S4 Table — Results are from tests completed upon study enrollment (baseline) before the Ob group began the exercise intervention. All values presented as mean ± standard deviation. Individual fatty acids are presented as µmol/l. Enzyme estimates are presented as ratios of fatty acids and are unitless. P-values are from a two-way ANOVA with sex and study group as the factors. Individual means were compared with Fisher’s least significant difference tests uncorrected for multiple testing. SCD1, stearoyl CoA desaturase 1. * different from females within group, p < 0.05; † different from NW group of the same sex, p < 0.05. The group with normal weight had 15 female and 20 male participants, while the group with obesity had 26 female and 30 male participants. (PDF) [file pone.0338547.s004.pdf]

**S4 Table. Fatty acids and estimates of lipid enzymes for females and males within each study group.**

| | NW Females | NW Males | Ob Females | Ob Males | P-value for sex | P-value for group | P-value for interaction |
|---|---|---|---|---|---|---|---|
| Eicosapentaenoic acid (20:5) | 0.47 ± 0.17 | 0.76 ± 0.95 | 1.62 ± 0.57† | 2.09 ± 1.54† | 0.101 | <0.001 | 0.692 |
| Linolenic acid (18:3) | 16.22 ± 8.62 | 12.93 ± 7.38 | 11.68 ± 3.69 | 13.22 ± 6.50 | 0.536 | 0.134 | 0.089 |
| Docosahexaenoic acid (22:6) | 4.17 ± 2.10 | 5.64 ± 4.46 | 9.22 ± 3.22† | 9.87 ± 3.29† | 0.158 | <0.001 | 0.589 |
| Arachidonic acid (20:4) | 2.79 ± 1.40 | 3.11 ± 1.70 | 3.61 ± 1.19 | 4.05 ± 1.33† | 0.212 | 0.005 | 0.837 |
| Linoleic acid (18:2) | 98.56 ± 32.61 | 90.21 ± 45.48 | 93.46 ± 29.43 | 101.91 ± 38.55 | 0.995 | 0.682 | 0.298 |
| Palmitoleic acid (16:1) | 18.98 ± 7.22 | 17.19 ± 11.74 | 19.9 ± 9.26 | 20.56 ± 10.09 | 0.797 | 0.320 | 0.567 |
| Oleic acid (18:1) | 181.00 ± 89.85 | 172.22 ± 106.62 | 168.73 ± 63.51 | 180.88 ± 69.52 | 0.924 | 0.918 | 0.553 |
| Elaidic acid (t18:1) | 10.18 ± 5.82 | 9.95 ± 6.27 | 11.64 ± 4.32 | 12.80 ± 4.85 | 0.684 | 0.060 | 0.542 |
| Myristic acid (14:0) | 11.00 ± 4.94 | 10.40 ± 6.71 | 7.90 ± 2.78† | 8.14 ± 3.19 | 0.851 | 0.006 | 0.662 |
| Palmitic acid (16:0) | 137.19 ± 59.72 | 129.34 ± 65.82 | 123.19 ± 41.10 | 133.62 ± 46.98 | 0.910 | 0.670 | 0.423 |
| Stearic acid (18:0) | 40.34 ± 12.58 | 40.03 ± 14.82 | 34.27 ± 8.90 | 36.84 ± 8.66 | 0.636 | 0.056 | 0.548 |
| SCD1(16:1/16:0) | 0.14 ± 0.02 | 0.13 ± 0.03 | 0.16 ± 0.04 | 0.13 ± 0.03† | 0.077 | 0.006 | 0.602 |
| SCD1(18:1/18:0) | 4.44 ± 1.13 | 4.07 ± 1.37 | 4.86 ± 1.31 | 4.82 ± 1.20† | 0.458 | 0.036 | 0.540 |
| Elongase (18:0/16:0) | 0.31 ± 0.06 | 0.33 ± 0.07 | 0.29 ± 0.6 | 0.29 ± 0.06† | 0.313 | 0.028 | 0.308 |
| Elongase (18:1/16:1) | 9.28 ± 1.48 | 9.28 ± 1.48* | 8.90 ± 1.72 | 9.35 ± 1.46† | 0.031 | 0.019 | 0.243 |
| Delta 6 desaturase (18:3/18:2) | 0.16 ± 0.03 | 0.14 ± 0.03* | 0.13 ± 0.02† | 0.13 ± 0.02 | 0.082 | <0.001 | 0.054 |

Results are from tests completed upon study enrollment (baseline) before the Ob group began the exercise intervention. All values presented as mean ± standard deviation. Individual fatty acids are presented as µmol/l. Enzyme estimates are presented as ratios of fatty acids and are unitless. P-values are from a two-way ANOVA with sex and study group as the factors. Individual means were compared with Fisher's least significant difference tests uncorrected for multiple comparisons. SCD1, stearoyl CoA desaturase 1. * different from females within group, $p < 0.05$; † different from NW group of the same sex, $p < 0.05$. The group with normal weight had 15 female and 20 male participants, while the group with obesity had 26 female and 30 male participants.
